# Biological Aspects of Inflamm-Aging in Childhood Cancer Survivors

**DOI:** 10.3390/cancers13194933

**Published:** 2021-09-30

**Authors:** Francesca Rossi, Alessandra Di Paola, Elvira Pota, Maura Argenziano, Daniela Di Pinto, Maria Maddalena Marrapodi, Caterina Di Leva, Martina Di Martino, Chiara Tortora

**Affiliations:** 1 Department of Woman, Child and General and Specialist Surgery, University of Campania “Luigi Vanvitelli”, Via L. De Crecchio 4, 80138 Napoli, Italy; elvira.pota@unicampania.it (E.P.); maura.argenziano@unicampania.it (M.A.); daniela.dipinto@unicampania.it (D.D.P.); mariamaddalena.marrapodi@studenti.unicampania.it (M.M.M.); caterinadl.94@gmail.com (C.D.L.); martina.dimartino@unicampania.it (M.D.M.); chiara.tortora@unicampania.it (C.T.); 2 Department of Experimental Medicine, University of Campania “Luigi Vanvitelli”, Via S. Maria di Costantinopoli 16, 80138 Napoli, Italy; alessandra.dipaola@unicampania.it

**Keywords:** childhood cancer survivors, inflamm-aging, frailty, immune system, oxidative stress, senescence, therapeutic strategies

## Abstract

**Simple Summary:**

Around 80% of children treated for childhood cancer become long-term survivors. Although chemotherapy and radiotherapy improve survival of these patients, they cause a low-grade chronic inflammation (inflamm-aging) which induces premature aging processes and vital organ failure, a condition known as frailty. Understanding frailty’s biological and molecular mechanisms and identifying inflamm-aging key biomarkers in childhood cancer survivors could be useful to facilitate the screening of comorbidities and to understand whether treatments, used to counteract inflamm-aging, may prevent side effects.

**Abstract:**

Anti-cancer treatments improve survival in children with cancer. A total of 80% of children treated for childhood cancer achieve 5-year survival, becoming long-term survivors. However, they undergo several chronic late effects related to treatments. In childhood cancer survivors a chronic low-grade inflammation, known as inflamm-aging, is responsible for frailty, a condition characterized by vital organ failure and by premature aging processes. Inflamm-aging is closely related to chemotherapy and radiotherapy, which induce inflammation, accumulation of senescent cells, DNA mutations, and the production of reactive oxygen species. All these conditions are responsible for the onset of secondary diseases, such as osteoporosis, cardiovascular diseases, obesity, and infertility. Considering that the pathobiology of frailty among childhood cancer survivors is still unknown, investigations are needed to better understand frailty’s biological and molecular processes and to identify inflamm-aging key biomarkers in order to facilitate the screening of comorbidities and to clarify whether treatments, normally used to modulate inflamm-aging, may be beneficial. This review offers an overview of the possible biological mechanisms involved in the development of inflamm-aging, focusing our attention on immune system alteration, oxidative stress, cellular senescence, and therapeutic strategies.

## 1. Introduction

One of the greatest medical successes over the past five decades is the improvement in survival among children with cancer. It is estimated that 80% of children treated for childhood achieve 5-year survival, becoming long-term survivors (>5 years) [1,2]. Although increased survival rates are encouraging, several late effects often accompany the advancements in childhood cancer patients’ treatment [3,4,5]. The chronic late effects may increase over time and influence the physiological aging process, resulting in a premature alteration of vital organ system function during adulthood and predispose individuals to a major risk to prematurely develop chronic age-related health conditions, frequent hospitalization, and early mortality.

This condition is known as frailty [6,7]. Frailty is a condition characterized by the onset of diseases related to aging and an increase in mortality; it can be observed both in older adults and childhood cancer survivors (CCS) [8,9]. In the elderly, frailty is influenced by lifestyle and genetics, while in young adult survivors of cancer it is more likely related to organ system damage following treatments [10,11]. Type of drugs, dosage, time of exposure, and extension of the irradiated area are all associated with the reduced fitness of long-term survival cancer patients. Exposure to oncogenic insults (chemo- and radiotherapy) leads to inflammation, the accumulation of senescent cells, and the increasing of reactive oxygen species (ROS) and DNA mutations [12,13]. Radiation exposure activates an immune response, determining the activation of macrophages and the recruitment of neutrophils and lymphocytes and pro-inflammatory mediators’ production in order to support anti-tumor activity [14,15]. After irradiation, chemotherapy, or Hematopoietic stem cell transplantation (HSCT) long-term immune system alteration could persist [16,17]. Some studies have shown that compared with controls, survivors who underwent HCT for a primary hematologic malignancy at age ≤21 had a similar BMI but a higher percent fat mass [18]. In particular, CCS show a high risk of long-term negative consequences after HSCT [19,20], among them metabolic syndrome [19,21,22,23]. In the general population, metabolic syndrome pathophysiology is related to lifestyle factors, like reduced physical activity and incorrect diet, which are responsible for an increase of BMI and, consequently, low-grade inflammation [20,24]. In cancer survivors, metabolic syndrome pathogenesis is not yet well known, and it is reported that it occurs in the absence of overt obesity [20,21]. Several studies reported that low-grade inflammation, caused by the cytokine release from abdominal fat, is related to metabolic syndrome in CCS of HSCT, as well as in the general population [20,23,24,25]. Furthermore, in HSCT survivors, other factors could contribute to inflammation: immune dysregulation and alloreactivity in the form of chronic GVHD [20]. Definitely, low-grade inflammation has a key role in the pathogenesis of metabolic syndrome after HSCT; in particular, there is an important relation between inflammatory molecule levels and the increased android/ginoid fat ratio, indicating that central fat accumulation is responsible for the increased levels of these molecules [23,26]. Inflammatory response alteration is closely related to the onset of dysmetabolism; indeed, it could alter insulin resistance, fat metabolism, and microvascular dysfunction [23,26]. Muhic et al. demonstrated that lifestyle factors and endocrine function alteration have a key role in frailty. It is known that total body irradiation (TBI) influences glucose and lipid metabolism, determining type 2 diabetes mellitus and glucose intolerance [20,27,28,29]. Accordingly, Muhic et al. found that TBI is a risk factor for the metabolic syndrome in HSCT long-survivors. Indeed, they found high levels of glucose in plasma in these patients [20]. The exact mechanism of TBI-induced negative consequences is not well known; it would appear that it causes mitochondrial damage, inducing hyperlipidemia and the alteration of fat storage [20,30]. Furthermore, it was demonstrated that TBI determines a decrease in testosterone concentrations and, consequently, an increase in central fat accumulation, responsible for the inflammatory cytokines release [28,31].

**Figure 1:**
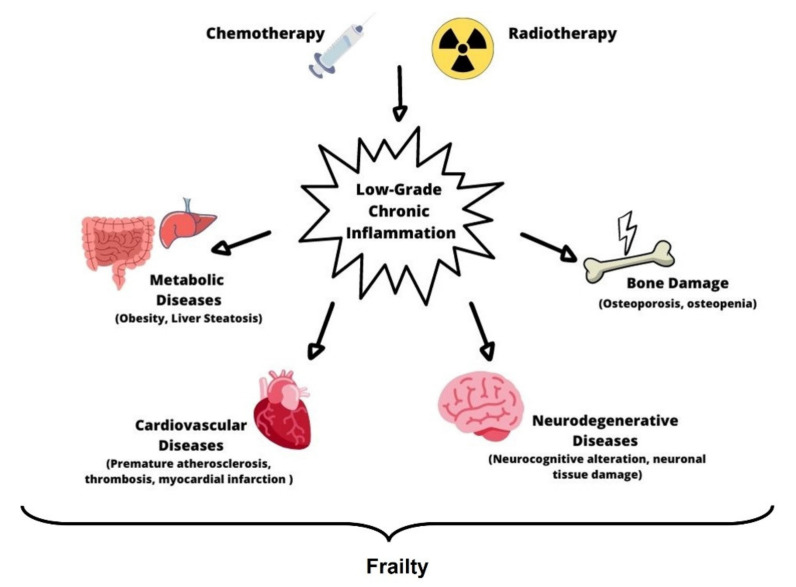
Frailty. Chemotherapy and radiotherapy cause a low-grade chronic inflammation in childhood cancer survivors, which is responsible for premature ageing processes and vital organ failure. This condition, known as frailty, determines the onset of several pathological conditions, such as metabolic diseases, cardiovascular diseases, neurodegenerative diseases, and osteoporosis.

Inflammation is responsible for the activation of two important cytotoxic mediators, ROS and reactive nitrogen species (RNS), which determine DNA damage [32,33]. Moreover, ROS/RNS stimulate the production of cytokines and adhesion molecules, and lymphocytes activation and proliferation [34]. The resulting condition of chronic low-grade systemic inflammation is named “inflamm-aging” [35]. Chronic inflammation shares several features of acute inflammation; however, it is persistent and of a low grade and induces responses that lead to tissue damage. Several mechanisms contribute to this condition: the continuous release of reactive molecules by infiltrating leukocytes; the cytokine production by both damaged non-immune and activated immune cells that regulate the inflammatory response [36]. Inflamm-aging is a systemic process that can involve any organ and tissue and predisposes the development of several disorders including osteoporosis, infertility, and metabolic and cardiovascular diseases [4,37,38,39,40] (Figure 1). The premature onset of these chronic diseases in CCS is particularly alarming considering that epidemiologic studies in survivors reveal that CCS aged 24 years have similar incidence rates of the onset of chronic diseases to their older family members aged 50 years [41]. Since the pathobiology of frailty among CCS is still unknown, the elucidation of frailty’s biological and molecular mechanisms in CCS and the identification of inflamm-aging key biomarkers are necessary both to facilitate screening of comorbidities in this population and to understand whether treatments, normally used to modulate inflamm-aging, may be in CCS. This review offers an overview of the possible biological mechanisms involved in the development of chronic systemic inflammation, focusing our attention on immune system alteration, oxidative stress, cellular senescence, and therapeutic strategies.

## 2. Inflamm-Aging and Immune System Alterations

**Figure 2:**
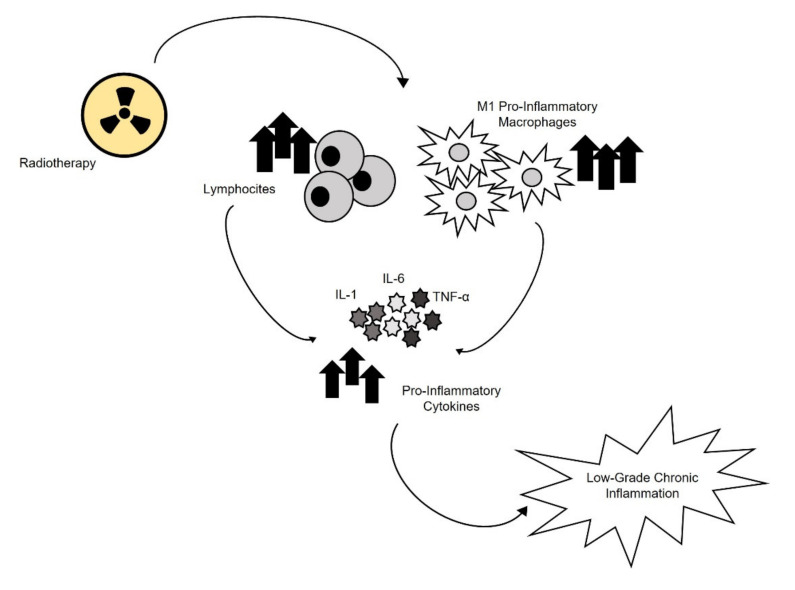
Radiation-induced immune response. Radiotherapy induces M1 pro-inflammatory macrophages and lymphocytes activation, leading to an increased production of pro-inflammatory cytokines, such as Interleukin (IL)-1, IL-6, and Tumor Necrosis Factor (TNF)-α, which contributes to the low-grade chronic inflammation, a typical condition of long-term cancer survivor patients.

Persistent alterations of the immune system in long-term childhood and adolescent cancer survivors result in a chronic low-grade inflammation similar to that observed with aging and contribute to a higher risk of secondary diseases [32]. A radiation-induced immune response, including macrophage activation and neutrophil and lymphocyte recruitment, leads to the production of pro-inflammatory mediators to support anti-tumor activity [14,15] (Figure 2). This inflammatory process could persist following chemo- and radiotherapy, or hematopoietic stem cell transplantation (HSCT) [42,43,44,45]. The mechanisms responsible for long-term chronic immune disturbances in cancer survivors and their potential consequences on survivors’ health still remain unknown. An immunosenescent phenotype, characterized by a decreased pool of naïve lymphocytes and the accumulation of memory and effector cells, could be attributed to T cells. In 2018, Daniel et al. identified a low-grade inflammation and an altered immune cell function in survivors treated with total body irradiation (TBI) and HSCT. Cancer treatments could induce long-term epigenetic changes in immune cells, in particular in T cell subsets distribution. They found a higher frequency of type 1 cytokine producing T cells in survivors and an over-activation of p38 and mTORC1 in these cells [34]. The over-activation of both p38 and mTORC1 is consistent with the greater frequency of Th1 cells and the higher levels of pro-inflammatory cytokines in survivors who received TBI/HSCT [34]. These changes in T cells might be involved in the perpetuation of the pro-inflammatory condition. Recently, Sulicka-Grodzicka et al. evaluated factors discriminating CS from controls, comparing selected biomarkers and lymphocyte subpopulations. They demonstrated that survivors had higher levels of C-reactive protein (CRP) and a shift towards activated CD8+CD38+ T cells [35]. CD38 is an important marker that regulates activation and proliferation of human T lymphocytes. T cells expressing high levels of CD38 have an enhanced cytokine production capability. Moreover, T cells of CS present a higher expression of CD28 than age matched controls. CD28 is essential in inducing T cell proliferation and survival and promotes the function of regulatory T cells, such as the production of interleukin-10 (IL-10) which is significantly elevated in young CD [46]. Ariffin et al. analyzed plasma inflammatory cytokines in 87 asymptomatic young adult survivors of childhood acute lymphoblastic leukemia (ALL) identifying high concentrations of IL-2, IL-10, and IL-17a. IL-17a, which is produced by activated Th17 cells, stimulates fibroblasts, endothelial cells, macrophages, and epithelial cells to release proinflammatory mediators, such as IL-1, IL-6, tumor necrosis factor alpha (TNF-α), nitric oxide synthase 2 (NOS-2), metalloproteases, and chemokines, resulting in the induction of inflammation [32]. Elevated plasma levels of IL-17a in the survivor group suggest microbial dysbiosis. In effect, many survivors experienced chemotherapy-induced mucositis, episodes of febrile neutropenia, and received several broad-spectrum antibiotics during the 2 years of leukemia therapy, which could alter the normal gut flora [32]. Accordingly, it has been demonstrated that the microbiome perturbation is a source of the chronic inflammation in patients with an immunodeficiency virus and a similar condition was reported also in a cohort of CCS [47]. IL-17a has effects on the cardiovascular (CV) system acting on vessel and cardiac cells, leading to inflammation, coagulation, and thrombosis. Several clinical studies have shown its involvement in the pathogenesis of CV disease, including premature atherosclerosis and myocardial infarction [48,49].

## 3. Inflamm-Aging and Oxidative Stress

**Figure 3:**
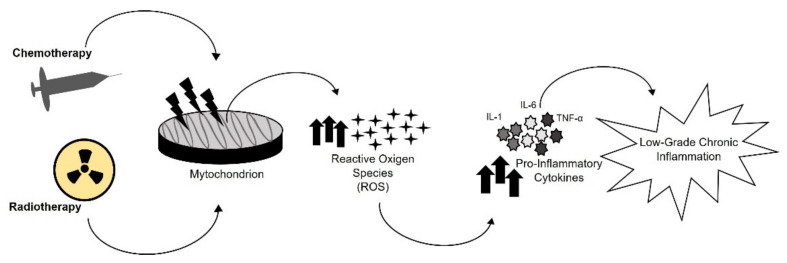
Oxidative stress. Chemotherapy and radiotherapy induced mitochondrial DNA damage, causing an increased production of Reactive Oxygen Species (ROS). ROS led to the activation of pro-inflammatory pathways, which induce inflamm-aging onset.

The hypothesis that oxidative stress stimulates inflamm-aging is well known and is supported by some evidence [50,51] (Figure 3). Oxidative stress could contribute to the pathogenesis of several diseases in CCS, leading to the activation of pro-inflammatory pathways [52]. Mitochondria are the major source of reactive oxygen species (ROS). Due to the proximity of mitochondrial DNA (mtDNA) to sites of ROS generation, mtDNA is prone to accumulating mutations after exposure to chemotherapy and radiation [53]. mtDNA mutator mice showed reduced levels of oxidative phosphorylation and developed a variety of several disorders, including osteoporosis, neurodegeneration, cardiomyopathy, diabetes, and muscle wasting [54,55,56,57,58]. In addition, inherited mitochondrial disorders are often accompanied by muscle atrophy and weakness, fatigue, and a decrease in exercise capacity, which are characteristics of the frailty phenotype described in CCS [8,59]. Moreover, the accumulation of damaged mitochondria limits the ability of muscle stem cells to sustain or to regenerate tissue, resulting in additional loss of muscle and, consequently, exacerbating existing frail health [60,61,62,63]. Future investigations are necessary to evaluate the relation between mitochondrial infidelity and accelerated aging in CCS. 

Protein glycation is another factor which contributes to inflamm-aging [64]. Advanced glycation end products (AGEs) are involved in the development and progression of inflammation [65]. AGEs’ high levels could be responsible for the alteration of function and structure of different proteins, such as fibrinogen, collagen, and low-density lipoprotein, leading to an inflammatory response [66]. The protein folding alteration induces an impairment of mitochondrial function, determining an increase of ROS production and a decrease of adenosine triphosphate (ATP) synthesis and of antioxidant intracellular activity [67,68]. Moreover, AGEs binding to their cell-bound receptor (RAGE) determines proinflammatory cytokines release and ROS production [69]. There is a crosstalk between AGEs increased levels and the onset of low-grade inflammation. Indeed, RAGE activation induced an increased expression of IL-1β and IL-17, which are important biomarkers of low-grade inflammation in CCS [70]. Accordingly, Felicetti et al. demonstrated that the TBI-exposed ALL survivors are characterized by a chronic inflammatory state probably due to AGEs’ increased levels; indeed, these patients present a seven-fold increase of AGEs compared to healthy controls and increased levels of CRP, IL-1β, and IL-17 [64]. Since AGEs are responsible for the onset of vascular damages and of endothelial cells activation, their serum concentration could be considered as a predictor of the cardiovascular disease severity in CCS [71].

Moreover, oxidative stress is not only a consequence of AGEs’ production, but also a mediator of their production; indeed, AGEs’ formation also occurs in the presence of oxidative stress [71]. It is known that the exposure to ionizing radiations or several chemotherapy agents increase ROS production and, consequently, oxidative stress, which could be perpetuated after the end of cancer therapies due to AGEs’ accumulation, known to be responsible for ROS production [72]. Oxidative stress-related cancer therapies could induce an increase in AGEs production, which in turn could cause inflammation, decrease antioxidant defenses, and induce ROS production, thus generating a vicious circle. Recently, several studies have suggested a role for AGEs also in second malignant cancer onset and progression in CCS. AGEs’ accumulation promotes the RAGE/RAS/NF-kB signaling activation, angiogenesis, and consequently tumorigenesis. A blockade of RAGE inhibits the angiogenesis of cancer reducing VEGF expression [73,74,75,76].

Moreover, mitochondrial stress can lead to the activation of stress responses through NLRP3 inflammasome formation, which induces IL-1β and IL-18 maturation, through its cleavage and secretion, and caspase-1 activation, suggesting the possible involvement of NRL3 inflammasome in second malignant cancer onset and progression in CCS [77].

The oxidative stress worsens the cognitive decline induced by chemotherapy in children with leukemia [78,79]. Corticosteroids and Methotrexate are the main drugs responsible for neurotoxicity in childhood ALL. In particular, Methotrexate causes an accumulation of homocysteine, a toxic amino acid, in blood and cerebrospinal fluid, causing neuronal tissue and vascular andothelium oxidative damage and, consequently, neurotoxicity [80]. Corticosteroids are known to influence mood and memory [81]. Cole et al. identified, as predictors of cognitive outcome in childhood ALL, several polymorphisms such as endothelial nitric oxide synthase (NOS3), hemo-Chromatosis (HFE), glutathione S-transferase pi (GSTP1), and the prostaglandin transporter (SLCO2A1). In particular, the NOS3 polymorphism has been identified as the variant mostly related to the neurocognitive outcome among the leukemia survivors analyzed. NOS3 has an important role in protection from oxidative damage. Homozygosity for the T allele in NOS3 results in decreased enzyme activity and in reduced capacity for oxidative stress protection. SLCO2A1 is not directly associated with oxidative stress [82]. The prostaglandin transporter encoded by this gene allows the movement of prostaglandins across the blood–brain barrier. Prostaglandins in CNS modulate many brain activities by regulating cerebral blood flow, synaptic transmission, neurotrophin production, angiogenesis, and also in chronic inflammatory processes generally associated with oxygen radicals’ production [83]. Therefore, functional variants influencing prostaglandin entry in CNS could alter the protective mechanisms against reactive oxygen species. Anthracycline is another important cause of morbidity in CCS [84].

Glutathione S-transferases (GSTs) are a class of phase II detoxification enzymes that induces the elimination of anthracyclines and plays a role in oxidative damage [85]. Singh et al. observed an association between the GSTμ1 (GSTM1) null genotype and anthracycline-related cardiomyopathy in anthracycline-exposed CCS. Moreover, they demonstrated a downregulation of GSTM1 gene expression in the peripheral blood as well as a reduced GSTM1 expression in human-induced pluripotent stem cell cardiomyocytes (hiPSC-CMs) generated from CCS who had anthracycline-related cardiomyopathy. This also provided a biological association between the GSTM1 null variant and anthracycline-related cardiomyopathy [86].

## 4. Inflamm-Aging and Cellular Senescence in CCS

Inflamm-aging in CCS is responsible for an early onset of aging, which induces molecular and cellular damage and the loss of physiological integrity [11,87]. In particular, cancer treatment exposures are involved in this process [87]. Aging causes the loss of physiologic capacity, determining an impairment of organ functions and death [87]. CCS are characterized by a premature loss of physiologic function closely dependent on anti-cancer treatments (chemo- and radiotherapy) [87], which induce not only the damage of malignant cells but also of non-malignant cells, such as neurons, cardiomyocytes, and skeletal muscle [11,87]. Cellular senescence, telomere attrition, DNA damage, and epigenetic alterations are the main processes involved in the acceleration of aging in CCS [87,88,89]. Telomeres, genetic elements located at the end of eukaryotic chromosomes, undergo a process to progressively attrition during each cell division, contributing to cellular senescence [32]. Telomere attrition is closely associated with chronic inflammation and, consequently, with an increased risk of the onset of age-related diseases, suggesting telomere length as an aging marker [32,90].

Aging determines an increase in the senescent cells rate [91], which shows a specific phenotype, known as senescence-associated secretory phenotype (SASP) [91,92,93]. Senescence progression is determined by the involvement of several factors, such as p16INK4A/retinoblastoma protein, p53/p21CIP1, and is also characterized by an alteration in genes expression, an arrest in cell cycle progression, apoptosis inhibition, and SASP [33,94,95,96,97]. SASP is mainly represented by pro-inflammatory cytokines, growth factors, chemokines, and other molecules which cause an alteration of the surrounding environment, influencing both nearby and distant normal cells [91,92,93] and, consequently, contributing to amplifying the negative effects of senescent cells on systemic function and surrounding tissues [98,99]. It has been reported that in CCS, SASP causes the onset of metabolic dysfunction, which contributes to an increased senescent cells rate [100,101].

**Figure 4:**
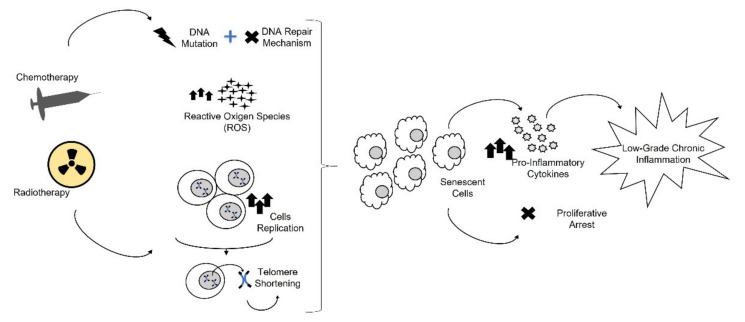
Senescence. Chemotherapy and radiotherapy cause DNA damage, impairment of repair mechanisms, Reactive Oxigen Species (ROS production), and continuous cell replication which is responsible for telomere shortening. All these consequences cause the onset of cellular senescence. Senescent cells are characterized by an increased release of pro-inflammatory cytokines and by the loss capability to grow and replicate, thus contributing to inflamm-aging.

Cellular senescence is a condition characterized by the loss of the cells’ capability to replicate or grow, caused by several factors [87]. This growth arrest is subsequently followed by permanent DNA alteration and by the impairment of repair mechanisms, thus making CCS more vulnerable to environmental exposures [11]. In detail, the exposure to chemo- and radiotherapy causes DNA damage, the over-expression of oncogenes, several mutations, continuous cell replication, and ROS production, which are all responsible for the onset of cellular senescence [96,100] (Figure 4). The proliferative arrest observed during cellular senescence is caused by telomere shortening after several cycles of cell division (replicative senescence) or by several stress events (stress-induced premature senescence, SIPS) [102]. Cells in stress-induced or in proliferative senescence and in quiescence show upregulated oncogenic miRNAs, which are involved in senescence and aging [11].

Mesenchymal stromal cells (MSCs) exert immune-suppressive and anti-inflammatory properties and are considered a promising source for treating autoimmune disease or counteract aging [101]. They are strongly damaged by senescence [101]; in particular, when MSCs become senescent, there is a reduction in their number and a loss of their immuno-modulatory and anti-inflammatory properties, thus contributing to exacerbating the inflamm-aging processes [101,103]. Indeed, it has been reported that aged MSCs release high levels of pro-inflammatory cytokines [104] and determine the macrophage phenotype switch from the M2 anti-inflammatory macrophages to the M1 pro-inflammatory ones [105], increasing the inflammatory state of senescence [101]. Since senescent MSCs are involved in the development and progression of inflamm-aging, they could be considered an effective target for anti-aging strategies [101].

Chemotherapy is mainly responsible for the onset of cellular senescence [91,99,106,107], contributing to accelerating the aging-like state and determining the appearance of senescence consequences, such as frailty and insulin resistance [91,92,93]. Indeed, it has been demonstrated that cranial radiation induced an increased expression of p16INK4a in a scalp biopsy specimen of children with acute lymphoblastic leukemia, confirming the role of cancer treatment in cellular senescence onset [108]. Hence, the research of innovative therapy to counteract these negative effects of anti-cancer drugs is needed [91,99,106,107]. Senolytics and SASP inhibitors are emerging promising drugs with a key role in preventing cellular senescence and its side effects [91,106,109]. In particular, senolytics degrade senescent cells, while SASP inhibitors counteract SASP effects [91,106,109], counteracting frailty, preventing cardiac and vascular disease risk, and determining a reduction of insulin resistance, of radiation therapy negative effects, and of osteoporosis [11].

## 5. Therapeutic Strategies to Counteract Inflamm-Aging in CCS

It is known that CCS may be prone to develop prematurely several age-related disorders, including osteoporosis, infertility, metabolic and cardiovascular diseases (CVD) [4,37,38,39,40].

Considering the severe effects of anti-neoplastic therapy in CCS, the investigation of cellular and molecular processes related to premature aging and chronic inflammation could be useful to discover novel therapeutic approaches to counteract the inflamm-aging in CCS.

Specific therapeutic strategies to counteract frailty in CCS are not yet well established, but it seems that pharmaceutical or nutraceutical agents and lifestyle improvement could ameliorate or prevent the frailty condition [8,110].

In particular, low-grade inflammation, caused by anti-neoplastic therapies, promotes pro-tumor microenvironment activation and, consequently, contributes to frailty [111,112,113]. Therefore, the identification of possible therapeutic interventions able to modulate inflammation in CCS could ameliorate their health condition [112].

### 5.1. Preventive Strategies to Counteract Inflammation: Lifestyle and Exercise

It was demonstrated that exercise could be an important non-pharmacological therapeutic strategy to counteract inflammation [112,114,115]. In particular, it could modulate immune system parameters [112], determining a reduction in tumor growth [112]. Increased pro-inflammatory cytokines levels are related to cancer outcomes. Indeed, in several cancer types, high levels of tumor necrosis factor (TNF) were detected [116,117]. Accordingly, it is reported that exercise is able to reduce TNF, IL-6, IL-8, and IL-2 levels in breast cancer [118].

In addition, immune function has a key role in cancer [112]. It was demonstrated that exercise training improves immune function in cancer [112,119], determining an increase of Natural Killer cells activity and lymphocyte proliferation [112,119]. Definitely, immune and inflammatory responses are modulated by exercise modalities [112,120]. Therefore, considering the beneficial effect of exercise in reducing inflammatory status and in modulating immune response, it could be considered an alternative non-pharmacologic therapeutic approach to counteract low-grade chronic inflammation in CCS.

Recently, it has been demonstrated that lifestyle is an important risk factor for metabolic and cardiac diseases in CCS [121,122]. Indeed, conducting a healthy lifestyle could prevent chronic diseases’ development, such as obesity and CVD, in CCS [123]. It was demonstrated that uncorrected lifestyle behaviors and unhealthy weight gain occur early in treatment in CCS and may persist beyond treatment completion and potentially into adulthood; therefore, a correct lifestyle should be initiated early in order to prevent all the consequent negative effects [124,125,126].

Moreover, it has been demonstrated that exercise could be safe and effective in the case of cardiac dysfunction in CCS [127].

Since CVD is closely related to inflammation, the use of anti-inflammatory drugs or drugs to counteract cardiovascular risk (hypertension, dyslipidemia) is proposed to treat high-risk patients in order to ameliorate long-term outcomes [10]. Moreover, in a recent study the therapeutic potential of the nutraceutical nicotinamide riboside (NR), a form of vitamin B3, has been evaluated in order to increase blood levels of nicotinamide adenine dinucleotide. Nicotinamide mononucleotide, derived from NR, improves cardiac function in a murine model characterized by an alteration of mitochondrial oxidative phosphorylation [128].

The main strategy to better manage aging consequences is aiming to ameliorate health behaviors [10,129]. In particular, it is advisable to lead a correct lifestyle: aerobic exercise and resistance training determines a reduction of fat volume and of pro-inflammatory cytokines production and concentration [130]. In the general population, exercises induce a reduction of chronic inflammation and of age-related telomere shortening, alter DNA methylation, and determine an increase of mitochondrial DNA [131,132,133,134,135,136,137]. In CCS, it is important to intervene with exercise both during and after anti-neoplastic treatment in order to counteract therapy-associated negative effects, thus ameliorating strength, walking speed, and lean mass [138,139,140,141,142]. Progression of these exercises should be modified basing on patients’ physiologic conditions and needs [143]. Indeed, patients with cardiac dysfunction show different responses to exercise based on the severity of their disease [143]. It has been reported that exercises in children with cancer or in CCS lead to an increase of strength, walking speed, and lean mass [127,138,139,141,142,144]. However, further studies are needed to better understand and clarify the most appropriate timing of the exercises’ application, or rather during therapy, immediately after therapy, or years later [87].

Since the increased risk of late effects in CCS is closely related to cancer therapies, changes in the standard treatment regimen have been made in order to maintain or improve cure rates and also to reduce the risk and the severity of late effects [145].

### 5.2. Biological Therapy and Immunotherapy to Counteract Inflammation

In recent years, new agents, more effective and less toxic than the canonical drugs used to counteract cancer progression, have been discovered [146]. In particular, in patients with sarcoma, lymphoma, and acute myeloid leukemia the simultaneous administration of molecularly target agents and conventional chemotherapy was studied [147,148,149]. For example, the combined use of the tyrosine kinase inhibitor, imatinib, and chemotherapy in Philadelphia-positive acute lymphoblastic leukemia induced an increase of percentage of 3-year even-free survival from 50% to 80% [150]. In many pediatric malignancies the administration of antibody-based therapy together with chemotherapy ameliorates outcomes of diseases. The use of several antibodies, such as brentuximab, gemtuzimab, and rituximab, is well documented to improve the onset of newly diagnosed or relapsed lymphomas and leukemias [151,152,153]. Further studies are currently underway for individuate therapeutics strategies that are more effective than canonical anti-neoplastic treatments and also without long-term side effects, which are unfortunately a consequence of chemotherapy and radiotherapy [154]. For example, the effects of genetically engineered chimeric antigen receptor (CAR) T cells has been the object of study in recent years [155]. Nevertheless, several long-term side effects are observed also after use of “targeted” therapies [156]. Considering this crucial aspect, it is necessary to perform a longitudinal systematic follow-up of children which received novel emerging therapies in order to verify whether these therapies could improve long-term outcomes compared with standard treatments [145,157].

Moreover, an emerging field is epitranscriptomics: it is based on the study of RNA modifications that do not affect the RNA sequence but affect functionality via a series of RNA binding proteins. Several kinds of epi-RNA modifications are known, such as 6-methyladenosine (m6A), 5-methylcytidine (m5C), and 1-methyladenosine. M6A modification is the most studied and could represent a potential RNA-modifying drug to treat leukemia [158].

### 5.3. Nutraceuticals’ Effect in Counteracting Inflammation

Recent studies have highlighted an emerging role of senolytics in inducing apoptosis of only senescent cells, but not of non-senescent cells [100,106,159,160,161,162]. Interestingly, six anti-apoptotic signaling pathways of senescent cells were discovered and it would seem that these pathways are involved in cancer cells’ defense against apoptosis [87,163]. Senolytics induce cancer cells apoptosis both in vitro and in vivo [100,159,160,161,162]. It has been demonstrated that they are able to counteract the negative effects of chronic diseases in animal models, such as osteoporosis, CVD, obesity, liver steatosis, metabolic alteration, frailty, muscle wasting induced by radiotherapy, and pulmonary fibrosis [137,159,162,164,165,166,167]. CCS show several of these chronic diseases and they appear to be responsive to these treatments; in particular, senolytics are able to reduce the premature aging phenotypes and all the consequent aging-associated diseases [8,100]. Moreover, another positive aspect about the use of these drugs in CCS consists in their ability to transiently interfere with the pro-survival pathways [87]. Additionally, in this case, it is necessary to establish the most appropriate timing of their administration, more precisely or after therapy subsequent to acute cellular damage, or after several years, when diseases appear [87].

## 6. Health and Management Tips for Childhood Cancer Survivors

The Childhood Cancer Survivor Study suggests that a 24-year-old childhood cancer survivor has the same risk of developing serious chronic conditions as their 50-year-old sibling [168]. CCS present deficits in attention, working memory, processing speed, and neurocognitive problems in everyday life [169], hence the need to integrate lifestyle interventions early in cancer care to promote healthy lifestyles. Studies suggested that the safety and feasibility of lifestyle interventions are significant if introduced while patients are receiving cancer treatment [170]. Most of the lifestyle interventions in CCS are focused on behavioral interventions in healthy aging like nonsmoking, caloric restriction, and physical activity [171]. A study demonstrated that both nutrition and physical activity are necessary to counteract the early onset of obesity and chronic diseases in CCS [172,173]. Few survivorship programs for childhood cancer have a specific focus on nutrition. Generally, to increase diet quality, to avoid excessive intakes of empty calories and sodium, and inadequate intakes of greens, beans, and whole grains are the recommendations for the survivors [174,175]. Family environment and parenting style play important roles in children’s dietary [176] intake and can become particularly important for children diagnosed with cancer at a young age. Very often, while the child is going through active cancer treatment, parents have permissive attitudes favoring unhealthy eating and sedentary behavior. Following treatment completion, parents find it difficult to reverse the unhealthy eating habits and sedentary lifestyle that have been established during the cancer treatment [177]. Therefore, it is important that parents have parenting skills and practices to facilitate healthy lifestyle changes. CCS show less interest in physical activity than healthy controls [178], thus they are more predisposed to poorer psychosocial welfare, greater somatic symptoms, and higher risk for secondary diseases [121,179]. Several observational and intervention studies suggest the positive correlation between physical activity and better neurocognitive functions in CCS [180,181]. Barlow-Krelina et al. identified an association of physical activity with neurocognitive problems many years post treatment, with fewer problems over time for survivors who participated in physical activity more constantly [171]. In particular, they observed improvements over 20 years post diagnosis, suggesting the continued benefits of physical activity even long after cessation of treatment [171]. This study is in accordance with Riggs et al. that demonstrated a positive impact of physical activity on cognitive outcome in pediatric brain tumor survivors after 5.25 years post treatment [182]. Adherence to lifestyle interventions is very difficult for CCS. The key to successful intervention is certainly to begin soon after treatment to establish healthy lifestyle customs before the onset of frailty or other chronic conditions. Parental involvement is an important aspect, as well as the oncology care team, in promoting healthy lifestyles for children in cancer care. Future lifestyle interventions developed in partnership with cancer survivors, caregivers, and health care providers will help ensure that the interventions address the needs of CCS and have the greatest impact.

## 7. Discussion

Current anti-neoplastic treatments improve the survival of children with cancer, so that 80% of children treated for childhood cancer will become long-term survivors (>5 years) [1]. Although chemotherapy promotes the survival rate for childhood cancer patients, unfortunately it also predisposes the onset of different late pathological conditions [3,4,5]. In particular, in childhood cancer survivors (CCS) a premature aging process is observed which induces the damage of vital organs and, consequently, the onset of chronic age-related diseases, such as osteoporosis, cardiovascular diseases, obesity, and infertility [4,37,38,39,40]. This condition is named frailty and is subsequent to a low-grade systemic chronic inflammation, inflamm-aging, caused by chemo- and radiotherapy insults [35]. Anti-neoplastic therapies are responsible for inflammation and for an increase of senescent cells, DNA mutation, and oxygen reactive species (ROS) production [12,13]. The accumulation of senescent cells and exposure to oncogenic insults (chemo-and radiotherapy) leads to inflammation, accumulation of senescent cells, and the increasing of DNA mutations and reactive oxygen species (ROS), which are involved in the stimulation of immune cells [14,15]. The continuous activation of the immune system in CCS causes chronic low-grade inflammation which predisposes to a higher risk of secondary diseases [32,183]. More specifically, chemo- and radiotherapy induce a persistent activation and recruitment of immune cells, such as lymphocytes and macrophages, determining the production of pro-inflammatory molecules, and amplifying the inflammatory response [14,15,42,43,45]. Oxidative stress has a key role in inflamm-aging, contributing to the onset of several diseases in CCS through the activation of pro-inflammatory pathways [52]. Since mitochondrial DNA (mtDNA) is close to ROS generation sites, it could undergo mutations after anti-neoplastic treatments [53]. Inflamm-aging causes an early onset of aging in CCS, which induces molecular and cellular damage and a premature loss of physiological function, determining an acceleration of aging [11,87]. During aging, an increase of the number of senescent cells [91] is observed, characterized by a specific senescence-associated secretory phenotype (SASP), mainly represented by pro-inflammatory molecules, which alters the surrounding environment and causes the development of metabolic dysfunction [91,92,93]. During cellular senescence, cells are characterized by an arrest of replication and grow, which causes both DNA alteration and impairment of repair mechanisms, thus making CCS more vulnerable to environmental exposures [11,87].

## 8. Conclusions

In conclusion, chemotherapy and radiotherapy predispose CCS to an increased risk of developing diseases secondary to frailty and inflamm-aging. An understanding of the molecular and cellular mechanisms underlying early aging and chronic inflammation could be an important step in the research of new therapeutic strategies to counteract the onset of diseases related to anti-neoplastic treatments. In recent years, new agents more effective and less toxic than the standard anti-neoplastic therapy are used to arrest cancer progression [146]. For example, antibodies-mediated therapy together with standard chemotherapy seems to improve cancer outcome and to reduce the several negative long-term side effects in CCS [150,151,152,153]. Furthermore, ameliorating health behaviors could be needed to contrast inflamm-aging-associated diseases [10]. Exercises are considered a crucial intervention to better manage all the early aging-dependent diseases in CCS [100,106,159,160,161,162]. Considering senolytics’ abilities to induce the apoptosis of senescent cells, they have been proposed to manage diseases related to inflamm-aging, thus reducing the premature aging phenotypes and all the consequent aging-associated diseases [8,162].

Certainly, other investigations are needed to better understand the biologic mechanisms underlying frailty and inflamm-aging in CCS in order to find other therapeutic strategies able to counteract and prevent all the disorders consequent to these conditions.
